# Effects of lifestyle on sexual function among postmenopausal women

**DOI:** 10.4314/ahs.v21i4.40

**Published:** 2021-12

**Authors:** Mona Rahnavardi, Zahra Bostani Khalesi, Sedighe Rezaie-Chamani

**Affiliations:** Social Determinants of Health Research Center, Guilan University of Medical Sciences, Rasht, Iran

**Keywords:** Lifestyle, Postmenopausal Women, Sexual Function

## Abstract

**Background:**

A healthy lifestyle has a key role in reducing health problems. Since one of the most common problems in Postmenopausal women has been sexual dysfunction (SD). The specific purpose of the present study was to identify the effects of health-promoting lifestyle (HPL) on sexual function among postmenopausal women.

**Methods:**

The present cross-sectional, descriptive, and analytical study was conducted on 405 Postmenopausal women aged 45–60 years, using the convenience sampling method.

Data collection was done using three questionnaires of demographic, health-promoting lifestyle profile-II (HPLP-II) and female sexual function index (FSFI). Data were analyzed in the SPSS-16 using Pearson's correlation coefficient. The statistical significance level was regarded as less than 0.05.

**Results:**

In general, the result of this study identified a 68% prevalence of SD among participants. The mean score obtained from the HPLP II was 2.27 (SD = 0.42), the highest score of its sub-scales was spiritual growth and the lowest score was physical activity.

The mean score of FSFI among the studied women was 23.16 (SD = 0.29), the highest score of six sub-scales was satisfaction and the lowest score was lubrication among participants. A strong correlation was found between the total FSFI scores, and spiritual growth (r=0.048), interpersonal relations (r=0.02), stress management (r=0.000), (p<0.0001).

**Conclusion:**

The results of the study revealed that a healthy lifestyle affects sexual function. Given that a healthy lifestyle is one of the most important ways to help women overcome SD, a healthy lifestyle promoting interventions necessary for Postmenopausal women.

## Introduction

Menopause is a critical period and an age-related phenomenon of life that associated with physical, mental and social changes^1^. There are many factors, including lifestyle, affect these changes^2^. Lifestyle as an important factor of health includes “day-to-day behaviors” and the individual functions in activities, job, diet, and fun^3^. A healthy lifestyle has a considerable impact on reducing physical and psychological diseases of Postmenopausal women^4^.

Literature Review on the main causes of morbidity and mortality demonstrated that factors of lifestyle are the main contributors to many major health problems and this is mainly true for the elderly^5^. Healthy behavior habits, such as regular physical activity, healthy eating and contributes to the prevention or delay of symptoms and complications of menopause in women such as hot flash and vaginal dryness^6^.

A healthy lifestyle is necessary for normal sex^3^. SD is the problem of most Postmenopausal women and it has a significant effect on marital satisfaction^7^. It can be said that SD may result in various marital problems^8^. To minimize these complications and more compatible with them, efforts should be made to promote Postmenopausal women's healthy lifestyle^9^.

Sexual functioning can be defined as a process of giving and receiving sexual pleasure, often in the context of interaction with a sexual partner^10^. A significant amount of studies has reported that women's sexual functioning significantly declines in the Postmenopausal period^6^,^9^,^10^,^11^. Large scale population-based studies shown that between 25% and 43% of the Postmenopausal women experience sexual disorders. Decreased sex drive is the most common symptom of female sexual dysfunction^6^. Jamali et al. reported prevalence rates of SD are 81.5% among Iranian menopausal women. The most prevalent problems were Dyspareunia, arousal, and lubrication^12^. There are a variety of risk factors for low sexual functioning among women. These risk factors include urogenital atrophy, vaginal dryness, and decreased tissue elasticity all of which can affect sexual behavior in women^6^. Other changes associated with menopause are including an impaired body image, decreased libido, decreased arousal, or decreased orgasm, which reduces sexual function^13^. Furthermore, other factors such as mental health, stressful life events and adverse, and lifestyle practices also affect women's sexual function^14^. Although, the relationships between some sexual problems and healthy lifestyles for Postmenopausal are not well reported in the literature. But, in limited studies, a relationship has been found between sexual problems and the lifestyle of Postmenopausal women. So that vaginal dryness is related to lifestyles such as diet or exercise, Postmenopausal women who were less physically active reported more complaints of vaginal dryness^15^. The similar findings were noted as women who vigorously exercised reported fewer vaginal symptoms^16^. In a study of Postmenopausal women, women who were obese reported more vaginal symptoms^17^. Also, despite the fact that menopausal women have difficulties in many aspects of their sexual life, including sexual function, health care providers often ignore these needs, and very little is known about the role lifestyle plays in the sexual function in Postmenopausal women. While understanding the healthy lifestyle and the relationship between a sexual function with this lifestyle can empower women to engage in a healthy lifestyle to improve sexual health. Therefore, this study was conducted with the aim of identifying the relationship between HPL and sexual function in Postmenopausal women.

## Methods

A cross-sectional, descriptive, and analytical study examined the relationship between healthy lifestyle behaviors and sexual function. Study participants were 405women aged 45–60 years who had experienced natural menopause. Natural menopause was defined as 12 consecutive months after the last menstrual period (1). A convenience sample was used in this study. The minimum sample size was estimated to be 384 participants based on Finley et al.'s study^18^ into the lifestyle Can Augment Female Sexual Well-Being. The expected power was calculated to be 0.95. The formula for determining sample size is:

r= 0.20

α= 0. 05→z1−α2= 1.96

1−β= 0. 95→z1−β= 1.645

A total of 405 samples was used for this study. The inclusion criteria were as follows: Iranian nationality; Residence in Rasht; able to read and understand Persian; >45 and <60 years-of-age; natural menopause (as determined after 12 successive months of amenorrhea); married and living their spouse, and having intercourse in the past month. Women who were unwilling to participate in the study; women with a history of hysterectomy; participants who had a chronic disease (hypertension, diabetes, female urge urinary incontinence, male low libido, and premature ejaculation); using sex hormone supplements or Phytoestrogens as a type of herbal medicines (current users); drug abuse (narcotics, smoking, drinking.), emotional illness, physical disability, cognitive disorders intense or prolonged mental distress and psychological disorders were excluded from the research. Also, all incomplete answers to questions were excluded from the study.

The tools used in the present study consisted of the demographic questions, FSFI, and HPLP II. A demographic instrument for collecting background (age, employment status, monthly income, educational status, the number of pregnancies, the number of children, the number of full-term delivery, and substance use) was developed for the study. In this study, the validity of the demographic and socioeconomic questionnaire was determined based on the content validity by 10 members of the faculty of Nursing and Midwifery, Guilan University of Medical Sciences.

The HPLP II questionnaire was developed by Walker et al^19^ that measures the extent to which participants practice positive health behaviors. It is a 52-item self-report questionnaire, a 4-point behavior rating scale containing. The sub-scales consist of physical activity (8 items); health responsibility (9 items); spiritual growth (9 items); nutrition (9 items); stress management (8 items); and interpersonal relations (9 items). Replies was on the Likert-type scale (1 = never, 2 = sometimes, 3 = often and 4 = routinely). The overall HPLP-II score, was calculated from a mean of the women's responses to all 52 items. The overall and mean sub-scale scores range was 1–4 and higher scores representative more HPL. The validity and reliability of Iranian HPLP-II scale was performed by Tanjani et al^20^. In the present study, Cronbach's coefficient α was estimated on the overall and sub-scale scores. Cronbach's coefficient α estimated the true reliability or internal consistency of a scale. The Cronbach's coefficient α was 0.943 for the HPLP II and for the sub-scales ranged from 0.793 to 0.872.

Female sexual dysfunction (FSD) assessed using FSFI offered by Rosen^21^. This instrument had 19 questions about sexual function in the last 4 weeks. Questions included: questions 1–2 were related to sexual desire; items 3–6 to sexual arousal; items 7–10 to sexual lubrication; items 11–13 to orgasm; items 14–16 to sexual satisfaction, and items 17–19 to pain assessment.

The reliability of the scale and sub-scales were obtained by calculating Cronbach's alpha coefficient estimated ≥at 0. 70 for all participants that showed good reliability^22^. Mohammadi et al confirmed that the Persian version of the FSFI is valid and reliable. The reliability of the Persian version of the questionnaire for each of the six sub-scale s was calculated by internal consistency using Cronbach's alpha. The correlation between questions in all sub-scale s was >0.61 which was accepted. The internal consistency of the whole scale was>0.85, which indicated good reliability. Furthermore, based on the sensitivity and specificity analysis, Mohammadi et al identified a score of 28 as a desirable cutoff for the diagnosis of women with and without sexual dysfunction^23^.

Consent was obtained from the HPLP II and FSFI designers by using the scales in this study. The researcher requested participants to indicate informed consent to take part in research through the consent form. Demographic data were analyzed using frequency numbers and percentages of participants. Tests of normality such as the Kolmogorov-Smirnv and Shapiro-Wilk statistics performed in order to meet assumptions for parametric statistical analyses. Based on the obtained results, Chisquare tests and Pearson correlation coefficient used. Statistical significance was P < 0.05.

## Results

The mean age of 405 participants and their partners were 56 and 59 years, respectively. The average marriage duration to date was 35 years. The majority of the participants (N = 57;41.6%) and their partners (N = 55;40.15%) had Diploma. Most of the respondents (N =386; 95.31%) were household and the remainder were employed. The monthly income in the majority of participants (N=134;33.08%) was 5 to 10 million IRR. Most of these participants (N =216;53.33%) had experienced 3 to 5 pregnancies. Over half (52.84%) of the participants reported 3 to 5 full-term deliveries (refer to Table 1).

**Table 1:** Distribution of Participants According to Demographic Features

Variable			(N =405) %
**Age, Mean^1^ (y) ± SD^2^**	**Women**	56 ±	6
	**Partner**	59 ±	8
**Duration of marriage, Mean (y) ± SD**			35±9
**No. of pregnancies**		<2	72(17.8)
		3–5	216(53.33)
		6–8	87(21.48)
		9–11	23(5.67)
		>12	7(1.72)
**No. of full-term deliveries**		<2	82(20.26)
		3–5	214(52.84)
		6–8	87(21.48)
		9–11	18(4.44)
		>12	4(0.98)
**No. of child**		<2	84(20.75)
		3–5	216(53.33)
		6–8	85(20.98)
		9–11	17(4.2)
		>12	3(0.74)
**Education Status**	**Women**	Primary or Secondary School	25(18.25)
		High School	39(28.46)
		Diploma	57(41.6)
		University education	16(11.67)
	**Partner**	Primary or Secondary School	29(21.16)
		High School	32(23.36)
		Diploma	55(40.15)
		University education	21(15.33)
**Employment Status**	**Women**	Unemployed	386(95.31)
		Employed	19(4.69)
	**Partner**	Unemployed	271(66.91)
		Employed	134(33.08)
**Monthly Income**		<500	48 (11.85)
		500–1	134 (33.08)
		1–1.5	92 (22.72)
		1.5–2	90 (22.22)
		>2	41(10.12)
**Substance use**	**Women**	No	403(99.5)
		Cigarettes	2(0.5)
	**Partner**	No	306(75.55)
		Cigarettes	77(19.01)
		Opium	21(5.19)
		Alcohol	1(0.5)

1 Mean scores

2 Standard Deviation

As it is evident in Table 2, this study found a positive correlation between the total FSFI scores, and spiritual growth (r=0.048), interpersonal relations (r=0.02), stress management (r=0.000), (p<0.0001). Also, there is a negative correlation between health responsibility and total FSFI scores (r= -0.053), desire (r= -0.062), lubrication (r= -0.094), satisfaction (r= -0.031), and pain (r=-0.095).

**Table 2:** The correlation between the FSFI and HPLP II sub-scales

FSFI Sub-scales HPLP II Sub-scales	Desire	Arousal	Lubrication	Orgasm	Satisfaction	Pain	Total scores
**Spiritual Growth**	0.076**	0.046***	0.047***	0.045***	0.089***	0.081***	0.048***
**Interpersonal** **Relations**	0.000*	0.061*	0.007*	0.051*	-0.005*	-0.038***	0.02***
**Nutrition**	0.045*	0.045***	0.038*	0.084***	0.052*	0.016*	0.088**
**Physical Activity**	0.077**	0.032*	0.033*	0.051	0.045**	0.036*	0.06*
**Stress** **Management**	0.082**	0.088***	0.000***	0.000***	0.000***	0.097***	0.000***
**Health** **Responsibility**	-0.062***	0.028*	-0.094***	-0.015	-0.031***	-0.095***	-0.053***
**Total scores**	0.079***	0.000***	0.089***	0.000***	0.000***	0.055*	0.000***

* *P* < 0.05

** *P* < 0.001

*** *P* < 0.0001

The HPLP-II scores ranged from 1.38 to 3.74 with a (M = 2.27, SD = 0.42). The average overall obtained from the HPLP II score indicating that survey participants ‘sometimes’ to ‘often’ engage in health-promoting behaviors. In the six of the HPLP II, the mean scores in individual spiritual growth (M = 3.12, Mean Range= 2.98 to 3.74); interpersonal relationships (M = 2.62, Mean Range=1.91 to 3.68); health responsibility (M = 2.27, Mean Range = 1.89 to 3.28); nutrition (M = 2.18, Mean Range = 1.78 to 3.52); stress management (M = 2.09, Mean Range = 1.38 to 2.90); and physical activity (M = 1.45, Mean Range = 1.13 to 2.75). Overall, the highest score of six sub-scales were spiritual growth and the lowest score was physical activity. The FSFI scores ranged from 1.2 to 36 with a (M = 23.16, SD = 0.29). In the six sub-scales of the FSFI, the mean scores were obtained in desire (M = 2.11, Mean Range= 1.94 to 3.87); arousal (M = 3.27, Mean Range=2.32 to 5.09); lubrication (M = 2.08, Mean Range = 1.63 to 3.25); orgasm (M = 3.02, Mean Range = 2.89 to 3.71); satisfaction (M = 3.67, Mean Range = 2.33 to 5.12); and pain (M = 3.52, Mean Range = 2.50 to 5.05). In summary, the highest score of six sub-scales was satisfaction and the lowest score was lubrication in participants.

For comparison of HPLP II scores between participants with and without FSD, we initially divided the participants into two groups: participants without FSD, including women with a total score of FSFI higher than 26.55 and the other group with a lower score. Based on current research, the prevalence of SD 68%. T-test showed that the total scores of HPLP II (P < 0.001) and the scores in all of its sub-scales were significantly higher in the group without FSD compared to those with FSD (refer to Table 3).

**Table 3:** Comparison of HPLP II scores between participants with and without FSD

HPLP II Sub-scale	Women with FSD (N =277) (Mean ±standard error)	Women without FSD (N =128) (Mean ±standard error)	*P* value*
*Spiritual Growth*	2.56 ± 2.2	3.68 ± 1.3	<0.001
**Interpersonal Relations**	2.06 ± 4.7	3.18 ± 1.8	<0.001
**Nutrition**	1.82 ± 1.8	2.54± 2.6	<0.001
**Physical Activity**	1.17 ± 3.3	1.73 ± 3.5	<0.001
**Stress Management**	1.92 ± 2.8	2.26 ± 2.4	0.04
**Health Responsibility**	1.87 ± 2.3	2.67 ± 3.1	<0.001
**Total scores**	2.02 ± 2.2	2.52 ± 1.3	0.02

**FSD**: defined according to FSFI total score ≤ 28

## Discussion

The main objective of the current study was to examine the relationship between HPL and sexual function in Postmenopausal women. Results suggested that there is a strong correlation between the total FSFI scores, and spiritual growth, interpersonal relations, stress management. The results suggest that overall sexual functioning is significantly related to lifestyle variables. This is consistent with Graziottin and Basson's study, in their study, have reported that sexual behaviors as a complex issue are influenced by lifestyle and interpersonal relationships^24^. A study by Nazarpour et al^25^ among 405 Postmenopausal women has reported those women with regular physical activity had better sexual activity than women with lower levels of physical activity. In general, participants with regular exercise have better lubrication and orgasm^23^. Also, Abedi's results indicated that is a linear relationship between the individual dimensions of HPL and those of sexual function except for physical activity and pain^26^.

The findings of Jamali et al. showed that 81.5% of the postmenopausal women had SD. The most prevalent sexual dysfunction was arousal disorder in Persians and Dyspareunia in Arabs^27^ and there were positive correlations between total scores FSFI and spiritual growth, interpersonal relations, stress management. The results of the study by Allen et al.^28^ indicated that physical activity was associated with a decrease in FSD. There was much evidence that a healthy diet associated with a decreased in FSD. Modification of lifestyle would appear to be an effective approach to declining the risk of FSD^29^.

The result of this study identified a 68% prevalence of FSD in participants. Similar results have been found in previous studies. However, the range was wide across the studies, but, numerous studies reported a high prevalence of SD among Postmenopausal women 6,10, 11. The prevalence of sexual dysfunction in the study of Nazarpour et al.^11^ Was reported to be 61%.

In the current study, vaginal dryness was the most common sexual complaint in participants. While numerous studies have reported desire problems as the most common complaint, followed by orgasm problems^29^.

According to the HPLP-II, The average overall obtained from the HPLP II score indicating that survey participants ‘sometimes’ to ‘often’ engage in health-promoting behaviors. The highest score of six sub-scales was spiritual growth and the lowest score was physical activity. It is essential to have a regular plan for physical activity in order to be healthy in the elderly^30^. A regular exercise plan will decrease the incidence of vaginal dryness and will also reduce sexual dysfunction that will appear with aging^31^. A study by Abedi in Iran showed that the highest score was for responsibility for health and the lowest was for physical activity. Results obtained by Abedi in terms of physical activity are consistent with our results^26^.

## Conclusions

The results suggest that lifestyle factors are significantly related to sexual functioning in menopausal women. Given the high prevalence of sexual dysfunction in Postmenopausal women, there seems to be a deep gap to achieve the desired sexual function in this age group. The implications of this study for health providers include the importance of routine assessment of menopausal women's sexual function and encouraging all clients, especially the elder to engage in health-promoting behaviors. The menopausal women's sexual function assessment should be comprehensive to ensure that all six sub-scales ‘ sexual unction on FSFI is considered as health care services are implemented.

In addition, further understanding and clarification of healthy lifestyle behaviors and the relationship of its sexual function by menopausal women can empower individuals to improve a healthy lifestyle.

There are several important limitations to this study. The use of a cross-sectional design does not allow for tracking symptom change over time and precludes assessment of the temporal relationship of the effects of lifestyle on sexual function.

Selection bias may have been introduced, as the subjects who elected to participate may differ from those postmenopausal women that chose not to participate. Therefore, findings from this study may not be representative of all postmenopausal women. Further, The sample for this study was one of convenience, which may influence the results.

Due to the complexity of sexual function among postmenopausal women, a mixed-method design, including a quantitative and qualitative study is recommended, to identify the effects of HPL on sexual function among postmenopausal women.

## References

[1] Burkman RT (2016). Berek & Novak's gynecology.

[2] Thornton K, Chervenak J, Neal-Perry G (2015). Menopause and Sexuality. Endocrinol Metab Clin North Am.

[3] Farhud DD (2015). Impact of Lifestyle on Health. Iran J Public Health.

[4] Heidari M, Ghodusi M, Rezaei P, Kabirian Abyaneh S, Sureshjani EH, Sheikhi RA (2019). Sexual Function and Factors Affecting Menopause: A Systematic Review. J Menopausal Med.

[5] Fitzgerald D, Litt J, Ciliska D, Delmore B, Butson T (1984). Health Consequences of Selected Lifestyle Factors: A Review of the Evidence. Can Fam Physician.

[6] Harder H, Starkings RML, Fallowfield LJ (2019). Sexual functioning in 4,418 Postmenopausal women participating in UKCTOCS: a qualitative free-text analysis. Menopause.

[7] Lett C, Valadares ALR, Baccaro LF (2018). Is the age at menopause a cause of sexual dysfunction? A Brazilian population-based study. Menopause.

[8] Waetjen LE, Crawford SL, Chang PY (2018). Factors associated with developing vaginal dryness symptoms in women transitioning through menopause: a longitudinal study. Menopause.

[9] Cuerva MJ, Gonzalez D, Canals M (2018). The sexual health approach in postmenopause: the five-minutes study. Maturitas.

[10] Træen B, Štulhofer A, Janssen E (2019). Sexual activity and sexual satisfaction among older adults in four European countries. Arch Sex Behav.

[11] Nazarpour S, Simbar M, Tehrani FR, Majd HA (2018). Quality of life and sexual function in Postmenopausal women. J Women Aging.

[12] Jamali S, Rahmanian A, Javadpour S (2016). Examining the sexual function and related attitudes among aged women: A cross- sectional study. Int J Reprod Biomed.

[13] Clayton AH, Valladares Juarez EM (2017). Female sexual dysfunction. Psychiatr Clin North Am.

[14] Sadock JB, Sadock AV (2019). Comprehensive textbook of psychiatry.

[15] Jiannine LM (2018). An investigation of the relationship between physical fitness, self-concept, and sexual functioning. J Educ Health Promot.

[16] Li S, Holm K (2003). Physical activity alone and in combination with hormone replacement therapy on vasomotor symptoms in Postmenopausal women. Western Journal of Nursing Research.

[17] Wekker V, Karsten MDA, Painter RC, van de Beek C, Groen H, Mol BWJ (2018). A lifestyle intervention improves sexual function of women with obesity and infertility: A 5 year follow-up of a RCT. PLoS One.

[18] Finley N (2017). Lifestyle Choices Can Augment Female Sexual Well-Being. Am J Lifestyle Med.

[19] Walker SN, Sechrist KR, Pender NJ (1987). The health-promoting lifestyle profile: Development and psychometric characteristics. Nurs Res.

[20] Tanjani PT, Azadbakht M, Garmaroudi G, Sahaf R, Fekrizadeh Z (2016). Validity and Reliability of Health Promoting Lifestyle Profile II in the Iranian Elderly. Int J Prev Med.

[21] Rosen R, Brown C, Heiman J, Leiblum C, Meston R, Shabsigh D (2000). The female sexual function index (FSFI): a multidimensional self-report instrument for the assessment of female sexual function. Journal of Sex & Marital Therapy.

[22] Wiegel M, Meston C, Rosen R (2005). The female sexual function index (FSFI): cross-validation and development of clinical cutoff scores. J Sex Marital Ther.

[23] Mohammadi KH, Heidari M, Faghihzadeh S (2007). The female sexual function index (FSFI): validation of the Iranian version. Payesh Journal.

[24] Graziottin A, Basson R (2004). Sexual dysfunction in women with premature menopause. Menopause.

[25] Nazarpour S, Simbar M, Ramezani Tehrani F, Alavi Majd H (2015). Exercise and sexual dysfunction among Postmenopausal women in Iran. sjsph.

[26] Abedi Parvin, Jorfi Maryam, Afshari Poorandokht, Fakhri Ahmad (2018). How does health-promoting lifestyle relate to sexual function among women of reproductive age in Iran?. Global Health Promotion.

[27] Jamali S, Javadpour S, Mosalanejad L, Parnian R (2016). Attitudes About Sexual Activity Among Postmenopausal Women in Different Ethnic Groups: A Cross-sectional Study in Jahrom, Iran. J Reprod Infertil.

[28] Allen MS, Walter EE (2018). Health-Related Lifestyle Factors and Sexual Dysfunction: A Meta-Analysis of Population-Based Research. J Sex Med.

[29] Malik E, Sheoran P, Siddiqui A (2018). Health-promoting behaviors and menopausal symptoms: An interventional study in rural India. J Mid-life Health.

[30] Alaeenejad A, Farahaninia M, Janmohammadi S, Haghani H (2017). Relationship Between Health-Promoting Behaviors and Quality of Life in Postmenopausal Women. JCCNC.

[31] Goldstein I, Kim NN, Clayton AH (2017). Hypoactive sexual desire disorder: International Society for the Study of Women's Sexual Health (ISSWSH) Expert Consensus Panel Review. Mayo Clinc Proc.

